# A Magnetic Metal Hard Mask on Silicon Substrate for Direct Patterning Ultra-High-Resolution OLED Displays

**DOI:** 10.3390/mi13070997

**Published:** 2022-06-25

**Authors:** Lin Chen, Xiuxia Wang, Yu Wei, Chenggang Zhou

**Affiliations:** Center for Micro and Nanoscale Research and Fabrication, Hefei National Laboratory for Physical Science at Microscale, University of Science and Technology of China, Hefei 230026, China; chenlina@ustc.edu.cn (L.C.); wxiuxia@ustc.edu.cn (X.W.); weiyu558@ustc.edu.cn (Y.W.)

**Keywords:** shadow mask, magnetic metal hard mask on silicon substrate, high PPI, high uniform pattern, OLED displays

## Abstract

With the development of virtual reality/augmented reality (VR/AR) display devices, the conventional fine metal mask is limited by the wet etch process, which no longer meets the demand for high pixels per inch (PPI) displays. We deposited a layer of magnetic metal on the silicon substrate by physical vapor deposition (PVD), and then developed a 2-inch, 3175 PPI magnetic metal hard mask on silicon substrate (MMS) through deep silicon etching and other micro-nano processing for patterning Organic Light-Emitting Diodes (OLED) displays, which can achieve smaller pixel size and higher PPI. MMS can not only solve the bottleneck problem of the traditional invar alloy shadow mask with low PPI, but also reduce the bending caused by the deformation of the silicon-based mask due to gravity, so that it achieves high PPI and higher uniformity in OLED displays.

## 1. Introduction

As consumers and the industrial and military markets move towards AR and VR applications, there is a pressing need for high resolution near-eye devices. To achieve a truly immersive experience, VR devices should at least have a pixel density of 2000 PPI to eliminate the screen door effect [1]. The evaporation size of organic light-emitting materials has become smaller and smaller in order to meet the demand for increasing resolution. Patterning of small-scale organic materials is a crucial step in the fabrication of VR devices.

**Figure 1:**
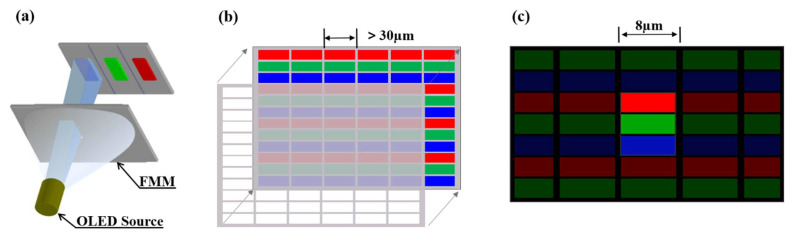
(**a**) Schematic diagram of OLED evaporation, (**b**) traditional OLED displays, (**c**) target OLED display.

Photolithography is usually not capable of patterning organic materials since the solvent used in the process may degrade the materials to be patterned. The conventional fine metal mask (FMM) such as Invar alloy mask is commonly used in the OLED display industry, as shown in Figure 1a [2]. Due to the limitations of the wet etching process with a pixel size of approximately 30 µm [3], as illustrated in Figure 1b, it is difficult to meet the pixel size ≤10 µm required by the high PPI AR/VR micro-display industry [1], as shown in Figure 1c. To overcome the limitations of the FMM technology, many processes have been proposed. Ink-jet printing [4,5,6,7] has been used but it suffers from potential drawbacks such as low throughput and non-uniform thickness. Other alternative patterning methods demonstrated include laser-induced thermal imaging [8,9] and laser-induced sublimation transfer [10,11]. With laser-induced thermal imaging, sharp edge patterns are difficult to achieve for polymer layers, and laser-induced sublimation can only be used to pattern small molecules [12].

In this paper, we have developed a silicon-based fine mask that can achieve high PPI and uniform evaporation. However, under the conditions of the influence of gravity; the bending of the silicon-based mask will affect the sizes of the vapor deposition pattern in different regions. We introduced a magnetic metal film in a new process, which not only acts as the etching barrier layer of the silicon substrate but can also use the upward magnetic force provided by the evaporation equipment to balance the hollow area bending of the silicon mask plate due to gravity.

## 2. Materials and Methods

**Figure 2:**
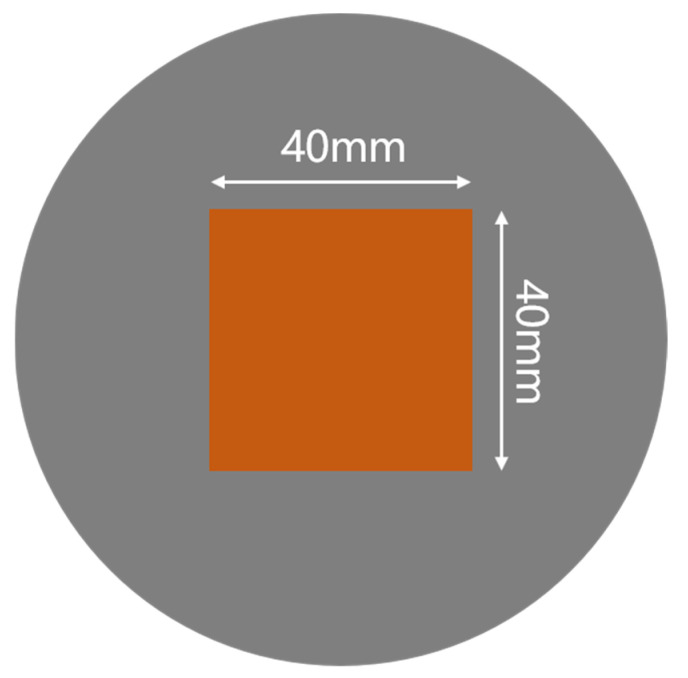
MMS dimensions.

The 2-inch MMS was fabricated on 4-inch Si wafer, as shown in Figure 2. The area of the free-standing magnetic metal hard mask on silicon substrate structure is 40 mm × 40 mm. The vertical thickness is approximately 20 µm, and pixel size is 4 µm × 4 µm.

**Figure 3:**
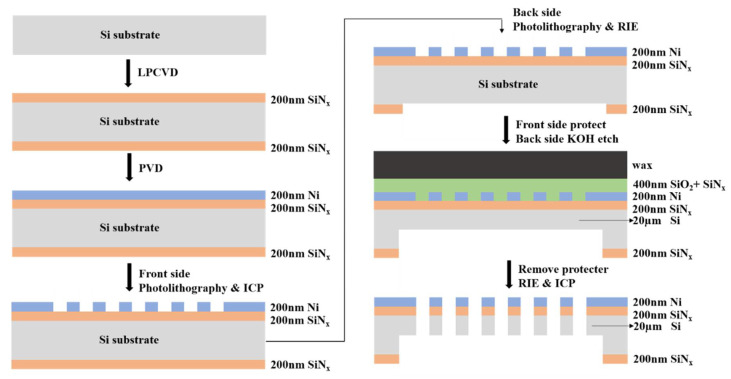
MMS process flow.

The process flow of MMS fabrication is illustrated in Figure 3. First, a layer of low-stress silicon nitride (SiN_x_) was deposited on both sides of the wafer by low pressure chemical vapor deposition (LPCVD) and then a Ni layer was deposited by Sputter or Ebeam at the front side. Photolithography and inductively coupled plasma etching (ICP) were first performed on the front side of the wafer to create an aperture array in the Ni layer. Photolithography and reactive ion etching (RIE) were acted on the backside of SiN_x_ layer to define the boundary of the free-standing area. After the front side protected by 200 nm SiO_2_ layer, 200 nm SiN_x_ and black wax, the exposed silicon on the backside was etched by 30% KOH solution at 70 degrees centigrade until left with 20 µm thick Si. Next, after the black wax was removed with CHCl_3_, the SiN_x_/SiO_2_ protective layer on the front side and the low-stress SiN_x_ not covered by Ni were etched with RIE. At the last step, the remaining 20 µm thick Si was etched with ICP, according to the shape of Ni with deep silicon etching process.

**Figure 4:**
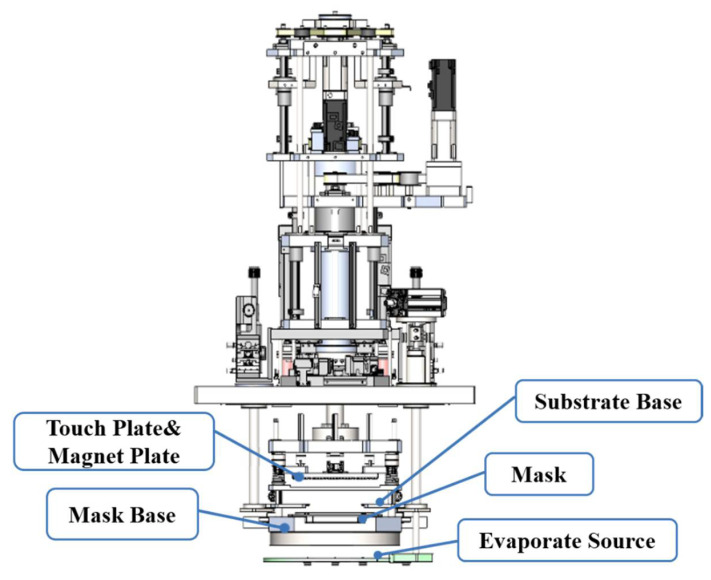
Working mechanism of evaporation mask.

The working mechanism of the evaporation mask is illustrated in Figure 4. The robotic arm transfers the substrate to the position to accurately align with the mask underneath. The contact plate above the substrate moves downward to compress the substrate and fix it. Meanwhile, the magnetic field to make the mask and the substrate fit more closely. As the thermistor heats the crucible below, the organic light-emitting material is evaporated and patterned on the surface of the inverted substrate.

## 3. Results and Discussion

### 3.1. Measures to Improve PPI

VR/AR products have an increasing demand for high PPI OLED displays. However, conventional fine metal masks such as the Invar alloy mask cannot further reduce the pixel size to less than 30 µm due to the limitation of the wet etching process accuracy. To solve this problem, we innovatively use silicon-based materials and the hard mask manufacturing process, which successfully promote the pixel size to less than 10 µm. However, there is a problem that the size of evaporated pattern differs between mask center and edge.

**Figure 5:**
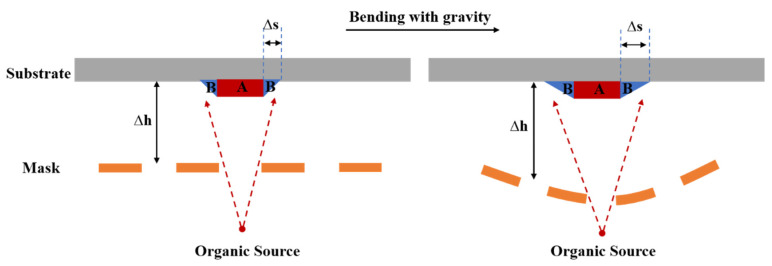
Diagram of shadow effects (∆S) with different gaps (∆h).

Taking the most common point evaporation source system as an example, a 10 μm~20 μm gap (∆h) is often set between the mask and the substrate to avoid particle contamination caused by contact. The exist of ∆h will cause the size of actual evaporation pattern (pattern A + pattern B) to be larger than the mask design value (pattern A), which is called the shadow effect [13], as shown in Figure 5. As the ∆h increases, the pattern B (∆s) increases, and the shadow effect becomes more obvious. The silicon-based hard mask will bend downwards under the influence of gravity, making the gaps different from mask center to edge, and the ∆s will change accordingly. Eventually, the CDs of organic light-emitting material evaporation pattern are nonuniform, resulting in an uneven display of the device.

### 3.2. Measures to Improve Uniformity

**Figure 6:**
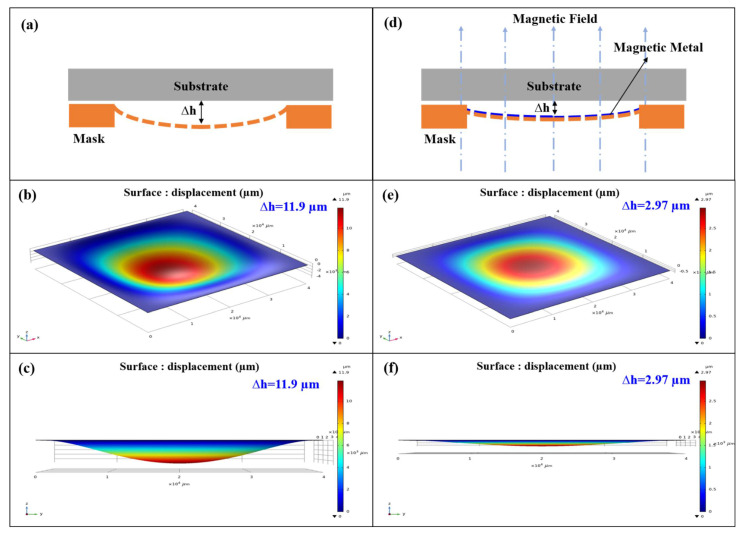
(**a**) Cross section schematic of Si based mask. (**b**,**c**) Simulated result of bending is 11.9 µm. (**d**) Cross section schematic of MMS. (**e**,**f**) Simulated result of bending reduces to 2.97 µm.

**Figure 7:**
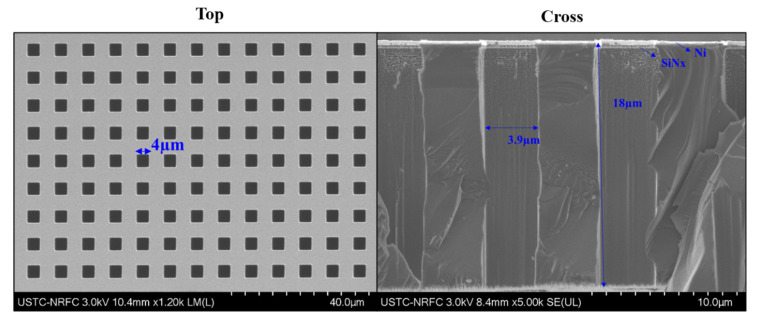
The SEM image of MMS.

The introduction of magnetic metal film solves this problem, which not only acts as the etching barrier layer of the silicon substrate in the deep silicon etching, but also provides upward magnetic force to balance gravity. In addition, considering the strength of the mask structure and the plugging rate of the through holes, the vertical thickness of suspended part in silicon-based hard mask is approximately 20 µm. We use finite element analysis to carry out simulation experiments. Under the condition of the influence of gravity and fixed constraints, the bending of the silicon-based mask(∆h) is 11.9 µm in Figure 6a–c. Furthermore, we introduced a magnetic metal film in new process, and the magnetic metal hard mask on silicon substrate has smaller bending (∆h = 2.97 µm). As the difference of gaps (∆h) from mask center to the edge gets shorter, the sizes of pattern B (∆s) get smaller, and the pattern sizes are more uniform, as shown in Figure 6d–f. Figure 7 is the SEM image of a magnetic metal hard mask on silicon substrate. In order to achieve the purpose of repeated or long-term use, MMS can be cleaned with ultrasonic vibration in organic solvent.

## 4. Conclusions

We have innovatively developed a 2-inch, 3175 PPI magnetic metal hard mask on a silicon substrate in order to meet the demand for high PPI and high uniformity of the micro-display in the VR/AR field. Thereinto, the silicon-based hard mask realizes high PPI, and the magnetic film layer which not only acts as the etching barrier layer of the silicon substrate, but also provides upward magnetic force to balance gravity realizes high uniformity display.

## Data Availability

Not applicable.
